# Effect of Cell Seeding Density and Inflammatory Cytokines on Adipose Tissue-Derived Stem Cells: an in Vitro Study

**DOI:** 10.1007/s12015-017-9719-3

**Published:** 2017-01-24

**Authors:** Panithi Sukho, Jolle Kirpensteijn, Jan Willem Hesselink, Gerjo J. V. M. van Osch, Femke Verseijden, Yvonne M. Bastiaansen-Jenniskens

**Affiliations:** 1 Department of Clinical Sciences of Companion Animals, Faculty of Veterinary Medicine, Utrecht University, Utrecht, The Netherlands; 2 Department of Otorhinolaryngology, Erasmus MC University Medical Center, Rotterdam, The Netherlands; 3 Department of Clinical Sciences and Public Health, Faculty of Veterinary Science, Mahidol University, Nakhon Pathom, Thailand; 4 Hills Pet Nutrition Inc, Topeka, Kansas, USA; 5 Department of Orthopaedics, Erasmus MC University Medical Center, Wytemaweg 80, 3015 CN Rotterdam, The Netherlands

**Keywords:** Adipose tissue-derived stem cell, Cell sheet, Seeding density, Inflammatory cytokine and paracrine ability

## Abstract

Adipose tissue-derived stem cells (ASCs) are known to be able to promote repair of injured tissue via paracrine factors. However, the effect of cell density and inflammatory cytokines on the paracrine ability of ASCs remains largely unknown. To investigate these effects, ASCs were cultured in 8000 cells/cm^2^, 20,000 cells/cm^2^, 50,000 cells/cm^2^, and 400,000 cells/cm^2^ with and without 10 or 20 ng/ml tumor necrosis factor alpha (TNFα) and 25 or 50 ng/ml interferon gamma (IFNγ). ASC-sheets formed at 400,000 cells/cm^2^ after 48 h of culture. With increasing concentrations of TNFα and IFNγ, ASC-sheets with 400,000 cells/cm^2^ had increased production of angiogenic factors Vascular Endothelial Growth Factor and Fibroblast Growth Factor and decreased expression of pro-inflammatory genes *TNFA* and Prostaglandin Synthase 2 (*PTGS2*) compared to lower density ASCs. Moreover, the conditioned medium of ASC-sheets with 400,000 cells/cm^2^ stimulated with the low concentration of TNFα and IFNγ enhanced endothelial cell proliferation and fibroblast migration. These results suggest that a high cell density enhances ASC paracrine function might beneficial for wound repair, especially in pro-inflammatory conditions.

**Electronic supplementary material:**

The online version of this article (doi:10.1007/s12015-017-9719-3) contains supplementary material, which is available to authorized users.

## Introduction

It is widely accepted that paracrine factors and cytokines from adipose tissue-derived stem cells (ASCs) can promote repair of injured tissue and/or improve the quality of tissues that are regenerated [38, 40, 41]. Traditionally, ASCs have been injected as cell suspension or have been combined with biomaterials before being delivered to injured tissue [2, 11, 19, 25, 29]. Recently, cell sheet engineering has been used to produce multicellular high density ASC-sheets that can be applied as a patch to injured tissue. Extracellular matrix, cell-cell and cell-matrix adhesions are preserved in engineered cell sheets thereby providing a niche and benefiting the attachment to tissue [39]. Several groups demonstrated that ASC-sheets are able to promote repair in different types of tissues such as skin [4, 16, 20, 39] and myocardium [13, 14, 23, 27, 37]. Formation of high-density ASC-sheets may influence the paracrine ability of ASCs [17] and subsequently affect their ability to promote tissue repair. Likewise, inflammatory cytokines present in injured tissues may impact ASCs function. Details about the effect of cell density and inflammatory cytokines on the paracrine ability of ASCs are largely unknown.

To elucidate these effects, we cultured ASCs in four different cell-seeding densities, with the highest density of 400,000 cells/cm^2^ resulting in actual sheets. Cells were cultured with and without tumor necrosis factor alpha (TNFα) and interferon gamma (IFNγ) to simulate an inflammatory environment. We demonstrated that ASCs cultured in a cell sheet at a density of 400,000 cells/cm^2^ have superior paracrine abilities when compared to ASCs cultured in lower cell densities in the presence of inflammatory factors. These findings offer opportunities to modify the beneficial effect of ASC-sheets for application in various tissues in the future.

## Materials and Methods

### Isolation and Characterization of ASCs

Human subcutaneous abdominal adipose tissue was obtained as waste material from female donors (age 46–52 years) with approval of the Medical Ethical Committee of the Erasmus Medical Center, Rotterdam (MEC-2014-092). ASCs were isolated as previously described [35]. Briefly, adipose tissue was digested with collagenase type I (Gibco, Life technologies, UK) for 1 h followed by centrifugation, washing to remove the oily layer including the adipocytes, and filtration through a 100 μm filter. Isolated ASCs were cultured in expansion medium (Dulbecco’s Modified Eagle Medium 1 g/l glucose (LG-DMEM, Gibco) with 10% fetal bovine serum (FBS, Lonza, Verviers, Belgium), 50 μg/ml gentamicin (Gibco), 1.5 μg/mL fungizone® (Gibco)at 37^ο^C in a humid atmosphere with 5% CO_2._ At 90% confluence, ASCs were subcultured with 0.25% trypsin EDTA (Gibco) and expanded starting at a density of 8000 cells/cm^2^ for use in the experimental set-up or stored in liquid nitrogen with 10% DMSO (Sigma-Aldrich, St. Louis, MO, USA) in expansion medium.

Cell surface phenotype was determined by flow cytometric analysis using mouse anti-human monoclonal fluorescently labeled antibodies directed against CD45-PerCp, CD14-FITC, CD34-APC, CD73-PE, HDL-DR-FITC (all BD Biosciences, San Jose, CA, USA), CD90-APC and CD105-FITC (R&D systems, Abingdon, UK) with dilutions according to manufacturer’s instructions. Unstained ASCs were used as control. After antibody staining, cell suspensions were washed twice with FACS flow (BD, Biosciences), resuspended in 200 μl FACS flow and directly analyzed on an eight colors FACSCANTO-II with FACSDIVA software (BD Biosciences) and FlowJo Software (Tree Star, Palo Alto, CA, USA).

To show multilineage differentiation capacity of the ASCs, adipogenic and osteogenic differentiation was performed in monolayer, and chondrogenic differentiation was performed in pellets as described previously [5]. To assess adipogenic differentiation, cells were stained with 0.5% Oil red O (Sigma) in isopropanol (Sigma). Osteogenic differentiation was assessed using Von Kossa staining with Thionin (Sigma). Pellets were fixed in 4% formalin overnight, paraffin embedded and sectioned (6 μm) before staining for Glycosaminoglycans (GAGs) with 0.4% Thionin solution (Sigma), to assess chondrogenic differentiation.

### ASCs Seeding and Culture Conditions

**Fig. 1:**
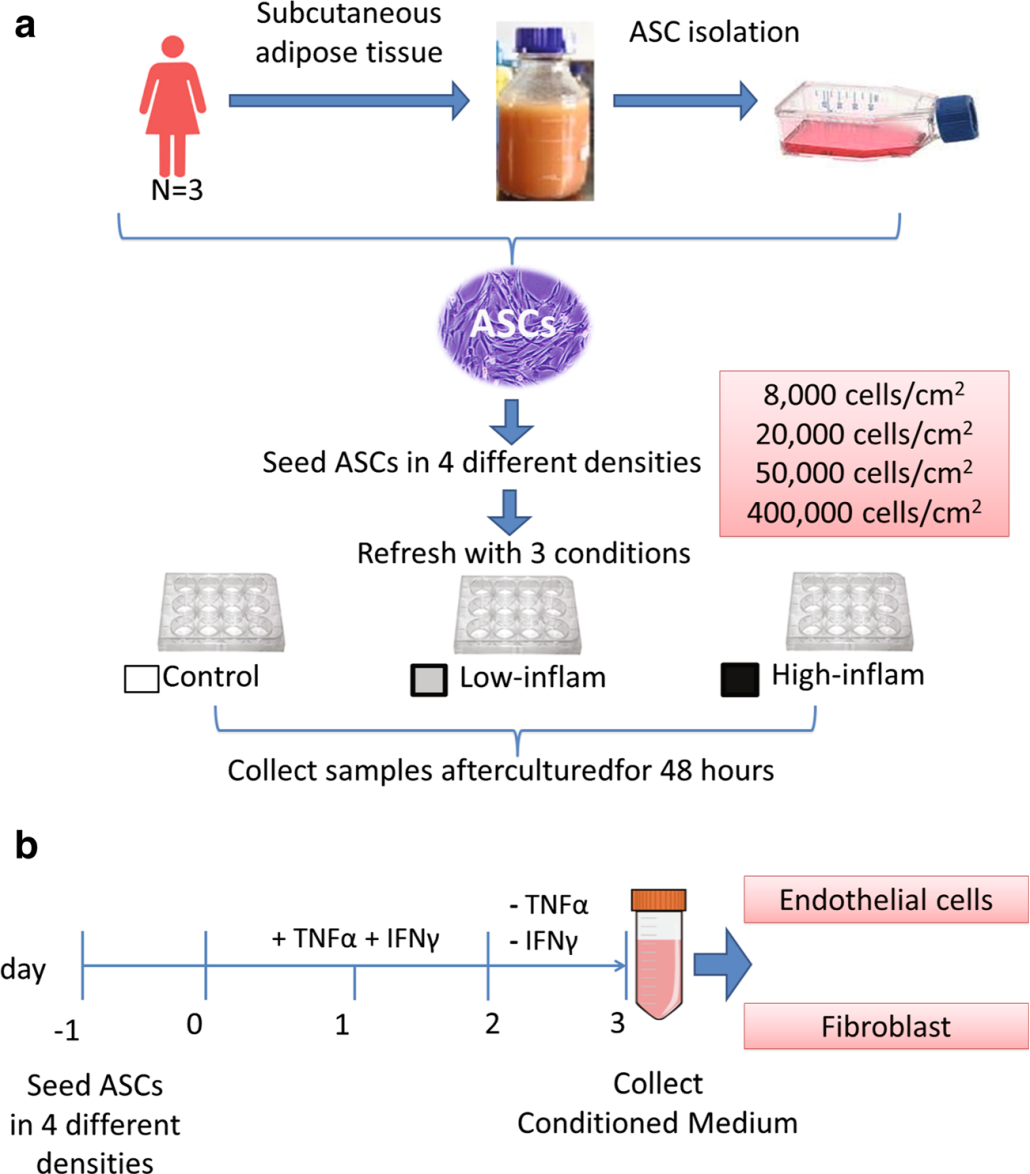
Experimental flowchart. (**a**) ASCs were isolated from 3 donors and seeded in 4 different densities and 3 different culture conditions for 48 h. (**b**) Conditioned medium collection. ASCs were cultured with TNFα and IFNγ for 48 h. Thereafter, medium was refreshed with culture medium containing no TNFα and IFNγ. Following 24 h ASCs-conditioned medium samples were collected for analysis

ASCs ≤ P4 were seeded in a 12-wells plate (Costar®, Corning Inc., Corning, New York) in 4 different densities: 8000, 20,000, 50,000 and 400,000 cells/cm^2^ in expansion medium overnight. We used the seeding density of 8000 cells/cm^2^ as a control in this experiment, since this seeding density is often used for expansion [7]. The seeding densities 20,000, 50,000 and 400,000 cells/cm^2^were selected based on previous studies examining cell sheets [10, 26, 36]. After overnight attachment, we refreshed the cells with LG-DMEM with 1% FBS, 50 μg/ml gentamicin and 1.5 μg/ml fungizone® (control condition). To induce inflammation, 10 ng/ml TNFα (PeproTech, Rocky Hill, New Jersey, USA) and 25 ng/ml IFNγ (PeproTech) -from now on referred to as low inflammatory condition-, or 20 ng/ml TNFα and 50 ng/ml IFNγ-from now on referred to as high inflammatory condition-were added as used earlier [30] and culture was continued for 48 h (Fig. 1). After 48 h of culture, medium and cells were harvested for analyses.

### ASC-Sheets Histology

ASC-sheets with a density of 400,000 cells/cm^2^ were harvested 48 h after culture in a temperature responsive plate (CellSeed, Tokyo, Japan). Reducing the temperature to room temperature resulted in detachment of cells allowing processing for analysis. For this, ASC-sheets were fixed overnight in 4% buffered formaldehyde followed by embedding in paraffin. Sections were stained with hematoxylin and eosin (Sigma, St Louis, Missouri and Merck, Billerica, Massachusetts, USA)

### Gene Expression Analysis

After 48 h of culture, ASCs were harvested with RLT lysis buffer (Qiagen, Hilden, Germany) plus 1% β-mercaptoethanol (Sigma-Aldrich). Total RNA was extracted using the RNeasy micro kit (Qiagen) with on-column DNA digestion. Total RNA was quantified using a NanoDrop™1000 spectrophotometer (Thermo Scientific, Wilmington, Delaware) according to manufacturer’s instructions and 200 ng RNA was reverse transcribed into complementary DNA (cDNA) using Revert Aid First Strand cDNA Synthesis Kit (ThermoScientific). The mRNA levels of prostaglandin synthase (*PTGS2,* HS01573474_g1), indoleamine 2,3-dioxygenase (*IDO1,* HS00158027_m1) and *FGF2* (HS00266645_m1) were analyzed with TaqMan® Gene Expression Assays (Applied Biosystem, Foster City, California, USA) according to the manufacturer’s instructions. Vascular endothelial growth factor A (*VEGFA)*, tumor necrosis factor alpha (*TNFA),* transforming growth factor beta-1 *(TGFB*) and glyceraldehyde-3-phosphate dehydrogenase *(GAPDH)* were analyzed with the quantitative polymerase chain reaction (Q-PCR) MasterMix Plus for SYBR®Green I dTTP (Eurogentec, Seraing, Belgium) with the following gene-specific primer sets: *VEGFA (*Fw 5′-CTTGCCTTGCTGCTCTACC-3′, Rv 5′- CACACAGGATGGCTTGAAG-3′)*, TNFA (*Fw 5′-GCC-GCA-TCG-CCG-TCT-CCT-AC-3′*,* Rv 5′-AGC-GCT-GAG-TCG-GCT-ACC-CT-3′*), TGFB* (Fw 5′-GTGACAGCAGGGATAACACACTG-3′, Rv 5′-CATGAATGGTGGCCAGGTC-3′, Probe: ACATCAACGGGTTCACTACCGGC) and *GAPDH* (Fw 5′-GTCAACGGATTTGGTCGTATTGGG-3′, Rv 5′-TGCCATGGGTGGAATCATATTGG-3′, Probe: TGGCGCCCCAACCAGCC). As *GAPDH* was stable between experimental conditions, we used *GAPDH* for data normalization. Real-time Q-PCR was performed with Bio-Rad CFX96 Touch™ Real-time PCR detection system and analyzed using CFX manager™ software (Bio-Rad Laboratories, Hemel Hempstead, UK). Relative expression was calculated according to the 2-ΔCT formula [31] using averages of duplicate samples.

### Analysis of Angiogenic Factors

Culture media were analyzed for the concentration of ASC secreted angiogenic factors; VEGFA and FGF2 using commercially available sandwich human VEGFA and human FGF basic DuoSet® ELISA kits (R&D systems). According to the manufacturers’ protocol, the optical density absorbance was determined at 450 nm with a reference wavelength of 540 nm in a VersaMax™ microplate reader. ELISA values are expressed as mean concentration of the total secreted factor per ml ± SD.

### L-Kynurenine Assay

Indoleamine-pyrrole 2,3-dioxygenase (IDO) is an enzyme that is able to inhibit T-cell proliferation via its metabolite L-kynurenine and thereby acts immune modulatory [30]. We determined the concentration of l-kynurenine as a measure of IDO activity in the culture medium as previously described by Leijs et al., 2012 [29]

### ASC Viability

Lactate dehydrogenase (LDH, Cytotoxicity Detection Kit, Roche, Mannheim, Germany) was measured to determine ASCs viability, according to the manufacturer’s protocol. Briefly, medium of ASCs was collected after 48 h of culture and centrifuged at 1500 rpm for 5 min to remove cells and debris. After that, 2% triton (Sigma-Aldrich) in LG-DMEM was added to the well and incubated for 2 h at 37^ο^C to damage all cells and served as maximum control in the assay to calculate the percentage of viable cells. One hundred microliter of medium and 100 μl lactate dehydrogenase reagent was mixed and incubated for 30 min in the dark at room temperature. The absorbance was measured with a VersaMax™ microplate reader (Molecular Devices, Sunnyvale, CA, USA) at 490 nm and a reference wavelength of 650 nm. Percentage of cytotoxicity relative to the maximum control was calculated according to the manual.

### ASC Conditioned Medium

To determine the effect of ASCs on fibroblast migration and endothelial cell proliferation, medium conditioned by ASCs in different densities in the presence of TNFα/IFNγ was made. The low inflammatory condition −10 ng/ml TNFα and 25 ng/ml IFNγ- is more close to physiologic concentrations of TNFα and IFNγ in injured tissue [33]. Additionally, gene expression profiles of ASCs were not different between the low and high inflammatory condition therefore medium was conditioned by ASCs cultured in different densities in the low inflammatory condition. Briefly, ASCs were seeded in densities of 8000, 20,000, 50,000 and 400,000 cells/cm^2^ and cultured in expansion medium overnight. After overnight culture, the expansion medium was replaced with LG-DMEM supplemented with 1% FBS, 50 μg/ml gentamicin, 1.5 μg/mL fungizone®, 10 ng/ml TNFα and 25 ng/ml IFNγ and cultured for another 48 h. Following stimulation with TNFα and IFNγ, the ASCs were washed with PBS and refreshed with LG-DMEM with 1% FBS, 50 μg/ml gentamicin, 1.5 μg/ml fungizone® but without TNFα and IFNγ and culture was continued. After 24 h, conditioned medium (CM) was collected and centrifuged at 1500 g for 5 min. The supernatant was stored in -80^ο^C until further analysis or used to culture endothelial cells and fibroblasts (Fig. 1b). Uncultured medium (LG-DMEM supplemented with 1% FBS) stored at -80^ο^C was used as control medium.

After media collection, each well was washed with PBS to remove non-attached cells, followed by addition of PBS to collect cells by scraping. Cells were digested overnight at 60^ο^C with 250 μg/ml papain (Sigma-Aldrich). The DNA amount was measured with the Cyquant® cell proliferation assay kit (Invitrogen) according to the manufacturer’s’ protocol (Sigma-Aldrich).

### Endothelial Cell Proliferation Assay

To test the effect of ASC-sheets on endothelial cell proliferation, human umbilical vein endothelial cells (HUVEC, Lonza) at P4 were seeded at a density of 5000 cells/cm^2^ in a 96-wells plate and in a 24-wells plate and cultured overnight in endothelial growth medium (EGM-2 bullet kit, Lonza). The next day, cells were starved with 0.5% FBS in LG-DMEM for 3 h. Then, HUVEC were refreshed with either control medium (LG-DMEM 1% FBS) mixed with EGM medium (1:1) or medium conditioned by ASCs mixed with EGM medium (1:1). After 0, 1, 2, 3, and 4 days endothelial cell proliferation and viable cell numbers were determined with the Cyquant® cell proliferation assay kit (Invitrogen) and MTT assay, respectively. Combining the results from these assays will allow to (indirectly) have an indication about the proliferation. According to the manufacturers’ protocol culture plates at -80^ο^C were frozen after removal of medium. The proliferation on each day was analyzed using known numbers of HUVEC as a DNA standard. At room temperature, 200 μl of CyQuant GR dye/lysis buffer was added to each well and incubated 5 min before reading the plate with the fluorescence microplate reader SpectraMax Gemini (Molecular Devices)

The MTT assay was based on the Mossman’s protocol [24] to check for metabolically active cells.

### Fibroblast Migration Assay

To investigate the migration of adult human dermal fibroblasts (HDFa, Gibco) in response to ASCs cultured in different densities, a scratch wound assay was performed with ASCs-conditioned medium. A scratch wound assay seems most suitable in representing wound healing in vitro, based on the cell migration pattern and direction of cell migration [15, 18]. HDFa *P* ≤ 6 were plated at 10,000 cells/cm^2^ in a 12-wells plate and allowed to adhere overnight in HG-DMEM supplemented with 10% FBS, 50 μg/ml gentamicin, and 1.5 μg/ml fungizone® to form a confluent monolayer. A linear wound was made in the cell layer of each well using a vertical scratch from a sterile 10 μl pipette tip after marking the scratch location on the bottom of the well. Cell debris was removed, followed by adding 100% ASCs-conditioned medium or control medium. Wound closure was captured using a 10× objective with phase contrast microscopy every 2 h from 12 to 24 h after scratching. The photos were blinded and analyzed with TScratch software (CSElab, Zurich, Switzerland) [9]. The percentage of wound closure was quantified and normalized to the freshly made scratch, which was considered as 100% open wound area.

### Statistical Analysis

Each experiment was repeated with 3 different ASCs donors and all experiments were performed in triplicate. Statistical analysis was done by one-way ANOVA for ASCs gene expression, protein analysis and viability followed by Bonferroni multiple comparisons. For the fibroblast migration assay and endothelial cell proliferation and viability, one-way MANOVA followed by Tukey HSD was used. Data were statistically analyzed with IBM SPSS statistical software 21 (SPSS, Inc., Chicago, IL, USA). A *p*-value < 0.05 was considered to be statistically significant. Data were expressed as mean ± SD.

## Results

### ASC Characterization and Morphology

**Fig. 2:**
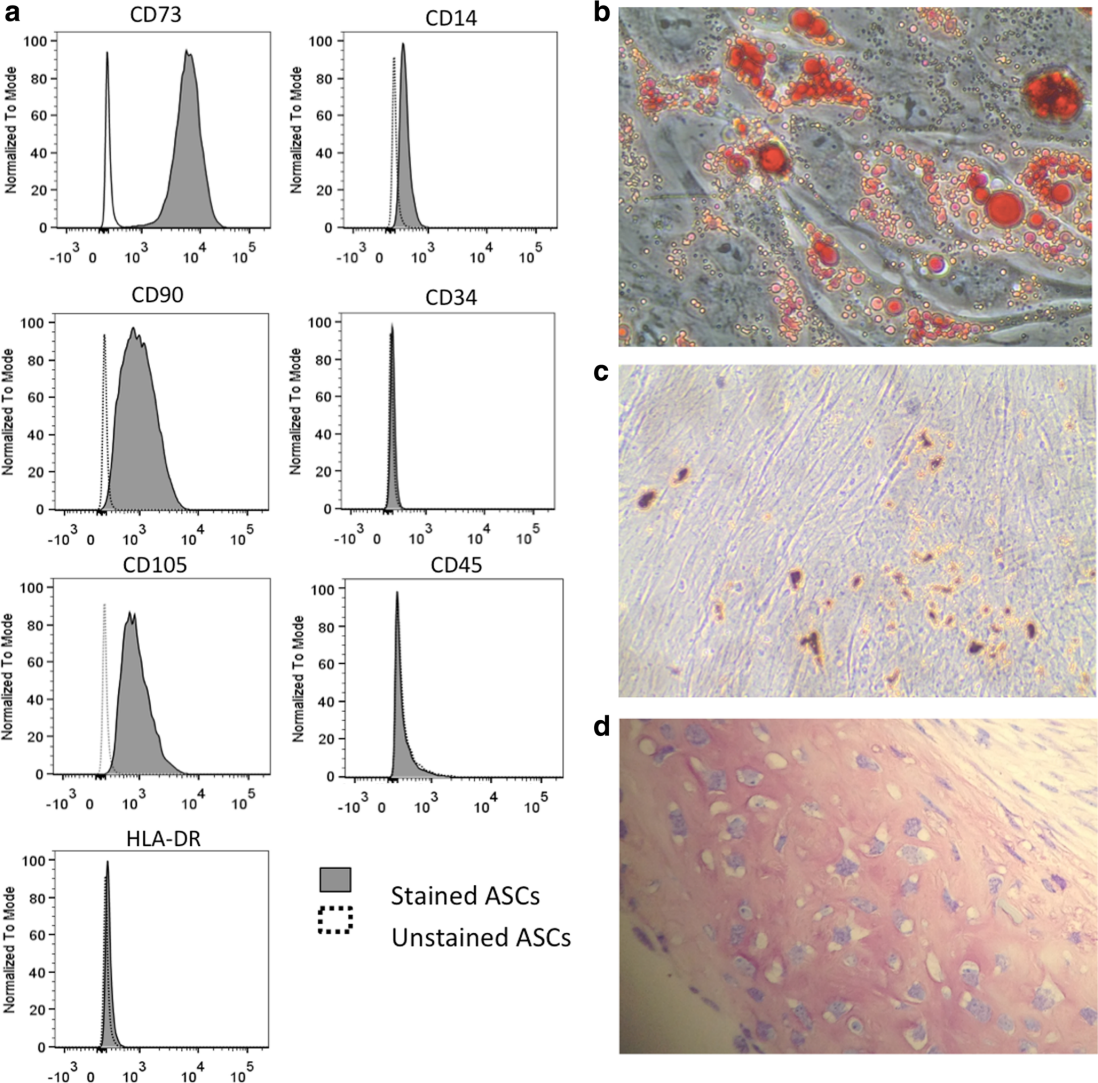
ASCs characteristics. (**a**) Cultured ASCs were positive for CD73, CD90, CD105 and had minimal expression of CD14, CD34, CD45 and HLA-DR as determined with flow cytometric analysis. (**b**) Adipogenic differentiated ASCs were positive for accumulation of lipid-containing droplets in the cytoplasm after staining with Oil Red O. (**c**) Osteogenic differentiated ASCs were positive for mineral deposition as stained with Von Kossa. (**d**) Chondrogenic differentiated ASCs pellets were positive for GAGs staining

**Fig. 3:**
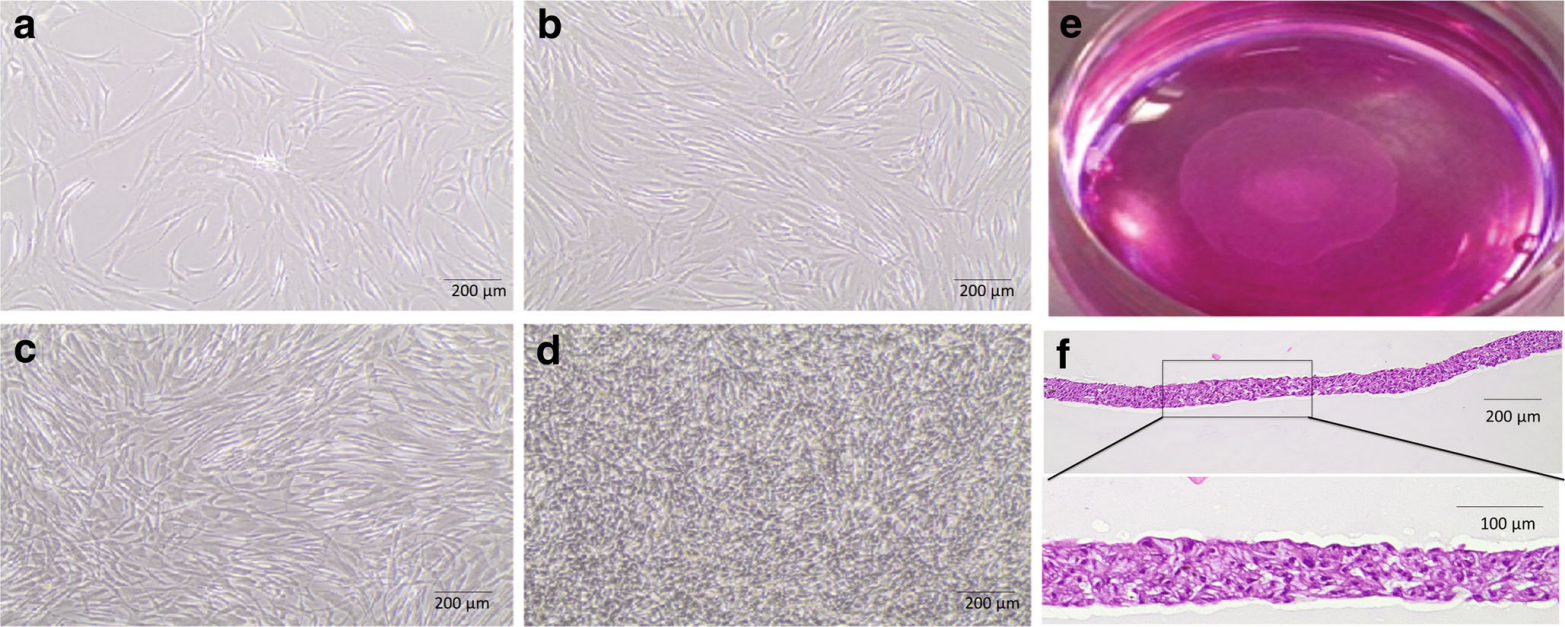
ASCs morphology in different seeding densities. a-d) ASCs morphology 48 h after seeding at (**a**) 8000 cells**/cm** ^**2**^, (**b**) 20,000 cells**/cm** ^**2**^, (**c**) 50,000 cells**/cm** ^**2**^ and (**d**) 400,000 cells**/cm** ^**2**^. (**e**) Image of a detached, floating ASC-sheet seeded at 400000 cells**/cm** ^**2**^. (**f**) Cross-section of a detached ASC-sheet stained with hematoxylin and eosin

To assess the phenotype of in vitro isolated and cultured ASCs, cell surface markers, morphology, and multilineage differentiation capacity were investigated. By FACS analysis (Fig. 2a), ASCs expressed mesenchymal stem cell markers such as CD73 (99.8% ± 0.1% of the total cell population), CD90 (90.3% ± 9.1%) and CD105 (87.4% ± 17.0%), but only minimal hematopoietic markers such as CD14 (5.7% ± 5.2%), CD45 (1.6% ± 0.6%) and CD34 (4.4% ± 3.9%) and human leukocyte antigen class II HDL-DR (2.3% ± 2.3%). Of notice, cell surface expression of CD73, CD90 and CD105 was a characteristic of the entire ASC population, thus operationally defining the homogeneity of the cells under study. As shown with the specific assays, ASCs also had differentiation capacities towards the adipogenic, osteogenic, and chondrogenic lineage (Fig. 2b). By phase-contrast microscopy, ASCs seeded at lower densities (8000–50,000 cells/cm^2^) displayed a spindle-shaped fibroblast-like morphology (Fig. 3a-c). When seeded at 400,000 cells/cm^2^, a multilayer, dense sheet of ASCs was seen (Fig. 3e, f).

### Effect of Cell Seeding Density and Inflammatory Cytokines on ASC Gene Expression

In the control condition -without TNFα and IFNγ- no significant differences were seen between the different cell seeding densities regarding gene expression of *VEGFA, TGFB, TNFA, IDO and PTGS2* **.**

The addition of TNFα and IFNγ to the culture medium increased the expression of *TNFA, IDO, PTGS2, VEGFA, and FGF2* for all cell-seeding densities whereas *TGFB* decreased in response to these pro-inflammatory cytokines*.* Interestingly, the addition of TNFα/IFNγ to 400,000 cells/cm^2^ ASC-sheets affected the expression of *TNFA, IDO* and *PTGS2* the least. In contrast, the expression of *VEGFA* and *FGF2* after addition of TNFα/IFNγ was affected the most in 400,000 cells/cm^2^ ASC-sheets**.**

**Fig. 4:**
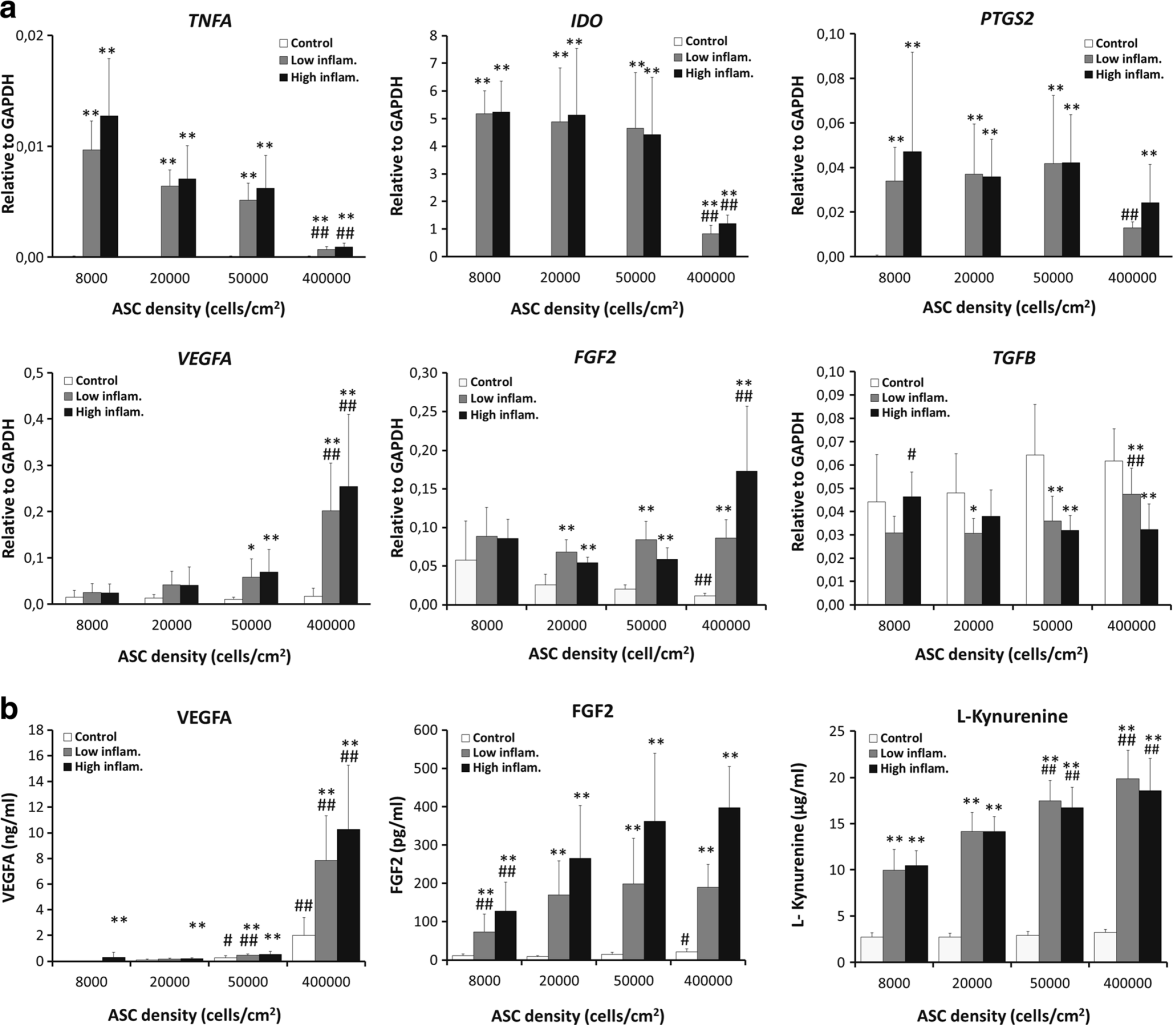
Gene expression patterns and cytokine production of ASCs seeded at different cell densities and in the presence of inflammatory cytokines- 10 ng/ml TNFα +25 ng/ml IFNγ (low inflammatory) and 20 ng/ml TNFα +50 ng/ml IFNγ (high inflammatory)- (**a**) *TNFA, IDO, PTGS2, VEGFA*, *FGF2* and *TGFB expression* relative to GAPDH and (**b**) VEGFA, FGF2 and L-kynurenine production. Each bar represents mean ± SD from 3 ASCs donors in triplicate (*P < 0.05, **P < 0.01 when compared with control condition within same density, # *P* < 0.05, ## *P* < 0.01 when compared within same culture condition)

No significant changes in the expression of *TNFA, IDO, PTGS2, VEGFA* and *FGF2* were noticed with increasing concentrations of TNFα and IFNγ in 8000, 20,000, and 50,000 cells/cm^2^. Increasing concentrations of TNFα and IFNγ increased *PTGS2, VEGFA*, *FGF2* and decreased *TGFB* expression in 400,000 cells/cm^2^ ASC-sheets (Fig. 4a)***.***

### Effect of Cell Seeding Density and Inflammatory Cytokines on ASCs Cytokine Secretion

We assessed whether increases in cell density led to changes in ASCs secretion of L-kynurenine, a metabolite of the immunomodulatory enzyme IDO and the angiogenic factors VEGFA and FGF2. Secretion of the IDO metabolite L-kynurenine was not affected by cell seeding density. In contrast, VEGFA secretion increased with increasing seeding density in the control condition from 34.7 ± 15 pg/ml at seeding density of 8000 cells/cm^2^ to 2005 ± 1394 pg/ml at seeding density of 400,000 cells/cm^2^. Likewise, the secretion of FGF2 in ASC-sheets seeded at 400,000 cells/cm^2^ was higher than by ASCs seeded at lower densities [21.6 ± 8 pg/ml at 400,000 cells/cm^2^ versus 11.7 ± 5 pg/ml at 8000 cells/cm^2^].

Similar to observed changes in gene expression, the addition of TNFα and IFNγ increased the secretion of VEGFA and FGF2. The highest levels of VEGFA, FGF2 and L-kynurenine levels were present in ASCs seeded at 400,000 cells/cm^2^ with a seemingly dose dependent effect of TNFα and IFNγ on VEGFA and FGF2 secretion (Fig. 4b).

### Analysis of ASC Viability

**Fig. 5:**
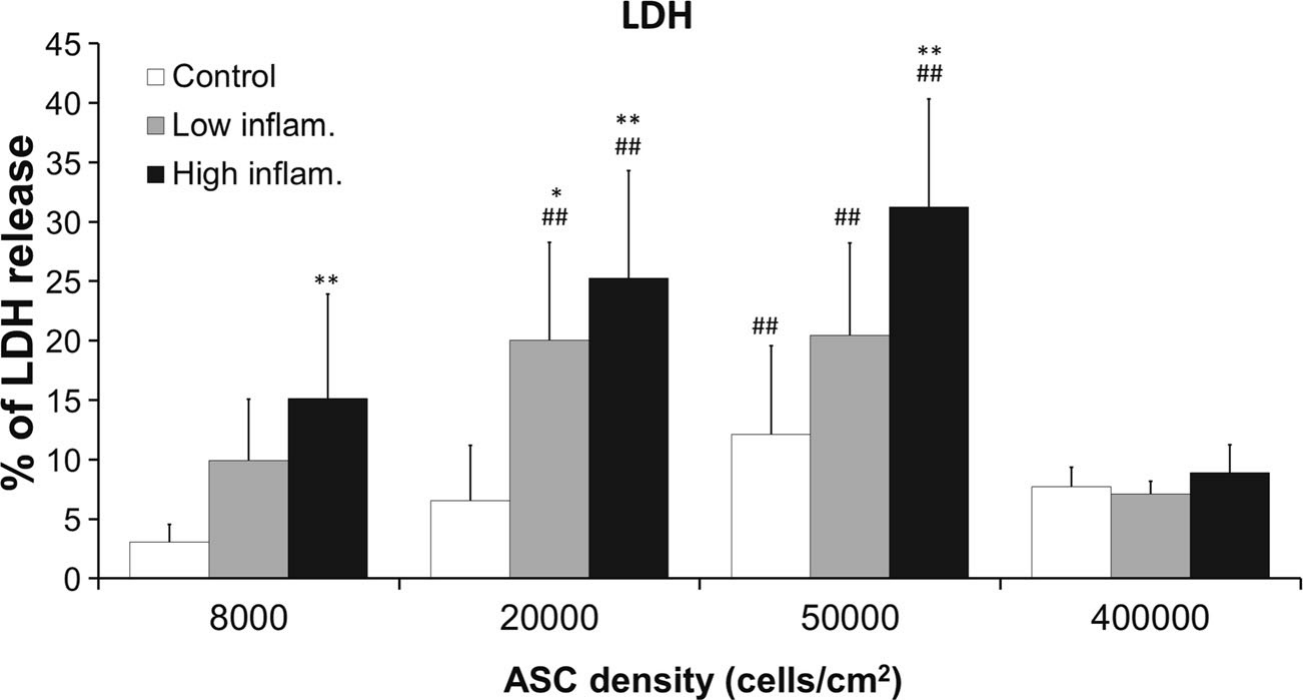
ASCs survival assessed by LDH release. ASCs were cultured in the presence of inflammatory cytokines −10 ng/ml TNFα +25 ng/ml IFNγ (low inflammatory) and 20 ng/ml TNFα +50 ng/ml IFNγ (high inflammatory)- and seeded at different densities. Each bar represents average percentage LDH release ± SD from 3 ASCs donors in triplicate. (*P < 0.05, **P < 0.01 when compared to control condition in same density, # *P* < 0.05, ## *P* < 0.01 when compared to control density (8000 cells/cm^2^) in same culture conditions)

As expected, an increase in cell seeding density resulted in decreased cell viability (as assessed by the LDH release) and a dose response was seen with increasing concentrations of TNFα and IFNγ in ASCs with 80,000, 20,000 and 50,000 cells/cm^2^ except for the highest density of ASCs (400,000 cells/cm^2^). However, seeding ASCs at 400,000 cells/cm^2^ resulted in no further decline in cell viability when compared to lower cell seeding densities. Moreover, at 400,000 cells/cm^2^ release of LDH remained low even though the concentration of inflammatory cytokines TNFα and IFNγ increased (Fig. 5).

### Effect of ASC-Sheet Conditioned Medium on Fibroblast Migration and Endothelial Cell Proliferation

**Fig. 6:**
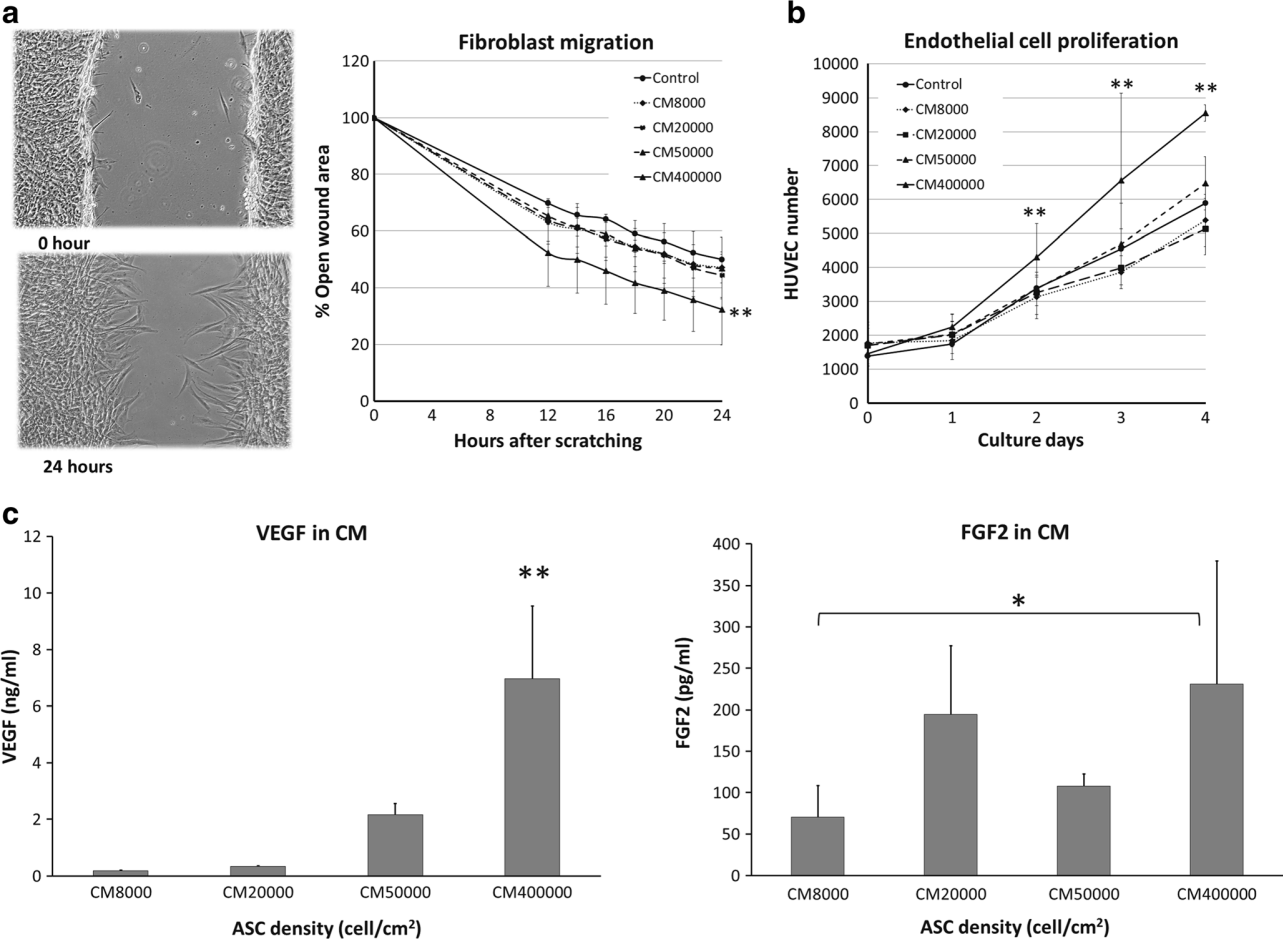
Assessment of HDFa migration and HUVEC proliferation when cultured in conditioned medium from ASCs seeded at different densities (**a**) Left panel; representative images of the scratch wound assay right after making the wound (upper panel) and after 24 h (lower panel). Right panel; the effect of ASCs conditioned medium on HDFa migration. Each mark represents the average percentage of open wound area ± SD; *n* = 3 donors in triplicate wells. (**b**) The effect of conditioned medium on HUVEC proliferation. Each mark represents average HUVEC number ± SD; *n* = 3 donors in triplicate wells (*P < 0.05, **P < 0.01 when compared between densities). (**c**) Average concentration ± SD of VEGFA and FGF2 in medium conditioned by ASCs from 3 ASCs donors 24 h after the removal of 10 ng/ml TNFα and 25 ng/ml IFNγ and prior to adding to HUVEC and HDFa

With the scratch wound assay with HDFa and medium conditioned by ASCs more wound closure after 24 h was seen in HDFa treated with conditioned medium from ASCs seeded at a density of 400,000 cells/cm^2^ (32.3 ± 15.9% open wound area) than in HDFa treated with control unconditioned medium (50 ± 13.8% open wound area) or in HDFa treated with conditioned medium from ASCs seeded at 8000 cells/cm^2^ (47.1 ± 10.6% open wound area) and 50,000 cells/cm^2^ (46.8 ± 8.4% open wound area; *p* < 0.05, Fig. 6a).

The effect of ASC seeding density on HUVEC growth and survival was also assessed. On day 2, 3, and 4, endothelial cell numbers were significantly higher in conditioned medium from ASCs seeded at 400,000 cells/cm^2^ than in conditioned medium from ASCs seeded at lower densities and control medium (Fig. 6b). HUVEC viability showed a similar trend; the percentage of metabolically active endothelial cells was highest in conditioned medium from ASCs seeded at a 400,000 cells/cm^2^ (Table 1).

**Table 1:** Percentage of metabolically active HUVEC after incubation with ASCs conditioned medium mixed with EGM (1:1)

ASC density (cells/cm^2^)	HUVEC culture (days)		
	2	3	4
8000	116 ± 14%	99 ± 2%	100 ± 4%
20,000	121 ± 22%	90 ± 1%	92 ± 17%
50,000	125 ± 23%	110 ± 10%	105 ± 17%
400,000	151 ± 29%^**^	164 ± 24%^***^	131 ± 42%^**^

Percentage of metabolically active HUVEC in response to conditioned medium of ASCs seeded at different densities and collected after culture in a low inflammatory condition. Metabolically active HUVEC were measured using the MTT assay. Effects of conditioned medium on metabolic active HUVEC are shown as a percentage in the corresponding unconditioned media. Percentages are expressed as mean ± SD; *n* = 3 donors in triplicate wells. *P < 0.05 **P < 0.01 *** *P* < 0.005 versus percentage of metabolically active HUVEC in unconditioned medium

The conditioned media that were used for these experiments had VEGFA and FGF2 levels in the same order of magnitude as when TNFα and IFNγ were still present, even though these stimuli were removed 24 h prior to harvesting the conditioned medium. In the case of VEGFA, the concentration increased with increasing ASC density. This effect was less clear for FGF2 (Fig. 6c).

## Discussion

Here we show that organized multilayer ASC-sheets with 400,000 cells/cm^2^ have superior paracrine characteristics when cultured in the presence of pro-inflammatory cytokines. The conditioned medium of these high-density ASC cultures are able to significantly enhance endothelial cell growth and fibroblast migration when compared to lower density ASC cultures.

In our study we used ASCs due to their therapeutic potential in regenerative medicine. For ASC isolation and characterization we used the accepted methods of isolation, plastic adherence, flow cytometric analysis, and multi-lineage differentiation. However, we cannot exclude the presence of small numbers of other cell types such as pre-adipocytes, endothelial progenitor cells, mast cells and others. Importantly, in this study we corroborate previous observations that ASCs at high seeding densities e.g. 400,000 cells/cm^2^ form multicellular ASC-sheets that can be harvested as patches and expand these observations by showing an association of these high-density ASC-sheets with an improved cell viability, increased paracrine potential and upregulation of genes involved in immunosuppression when exposed to pro-inflammatory cytokines.

In line with our expectations, increasing ASC seeding density decreased cell viability. However, increasing seeding density from 50,000 cells/cm^2^ to 400,000 cells/cm^2^ showed no further decline in cell viability. Additionally, *TNFA* gene expression decreased in response to increasing cell density under inflammatory conditions.

Previous studies have shown that pro-inflammatory cytokines as TNFα and IFNγ can stimulate mesenchymal stem cells to produce more TNFα, COX2 (encoded by *PTGS2*), and IDO and less TGFB [6, 30]. IDO is an enzyme that is able to inhibit T-cell proliferation via its metabolite L-kynurenine and thereby acts immunomodulatory [32]. Similar to these previous reports, when we stimulated ASCs with TNFα and IFNγ, *TNFA*, *IDO*, and *PTGS2* expression was higher than in unstimulated ASCs. Interestingly, the effect of pro-inflammatory stimulation by TNFα and IFNγ is less when ASCs are cultured in high density 400,000 cells/cm^2^ ASC-sheets. These high density ASC-sheets seem to be less influenced by inflammation. Moreover, increasing cell density and increasing concentrations of TNFα and IFNγ resulted in the secretion of more VEGFA, FGF2, and L-kynurenine, emphasizing the potential benefit of these high density ASC-sheets for regenerative medicine purposes, even in the presence of inflammation.

We chose to show the secretion of VEGFA, FGF2 and L-kynurenine as total protein production since the actual amount of protein will exert its beneficial therapeutic effect in vivo, irrespective of how much is produced per individual cell. However, when we correct VEGFA secretion for cellular DNA, the secretion of VEGFA per cell is still higher in the highest density ASC-sheets (Supplementary data Fig. 1).

We confirm previous observations that ASCs seeded at higher density upregulated VEGFA gene expression [17]. Hsiao et al., 2013 [12] and others [3, 28] showed enhanced angiogenic paracrine activity of hypoxic ASCs in vitro. Following up on these observations the higher levels of VEGFA secretion in our multilayer 400,000 cells/cm^2^ ASC-sheets might be due to some level of hypoxic stress.

The above-described results suggest that ASC-sheets with 400,000 cells/cm^2^ may have a higher ability to promote tissue repair than ASCs seeded at lower densities, especially under inflammatory conditions. To investigate this further, we obtained conditioned medium from ASCs seeded at different seeding densities and cultured in the presence of TNFα and IFNγ and found that conditioned medium from 400,000 cells/cm^2^ ASC-sheets significantly enhanced fibroblast migration and endothelial cell proliferation as two important processes in wound healing [8, 22, 34] compared to control medium and conditioned medium from ASCs seeded at lower densities. Since VEGFA secretion was significantly increased in these ASC-sheets compared to lower density ASCs cultures, it is possible that this growth factor contributed to the enhanced fibroblast migration and increased endothelial cell proliferation. However, besides VEGFA and FGF2 that we measured in our conditioned medium, the influence of other soluble factors in these processes is also very likely. Among many others, these factors could include TGFβ, hepatocyte growth factor and multiple interleukins which are also secreted by ASCs [41], but also the bioactive lipid sphingosine-1 phosphate which is known as an pro-angiopoietic factor [1].

Taken together, our data demonstrate for the first time that ASCs seeded in high-density cell sheets are beneficial for fibroblast migration and endothelial cell proliferation in vitro. This is in agreement with studies directed at in vivo use of ASC-sheets. In these studies, the use of high density ASC-sheets was linked to enhanced tissue healing and angiogenesis [10, 13, 14, 21]. In addition, our data indicate that stimulating ASC-sheets with pro-inflammatory stimuli in vitro may be beneficial regarding tissue vascularization. However, the possibility of improving tissue vascularization by culturing ASC-sheets with pro-inflammatory stimuli in vitro needs to be confirmed by further in vivo studies which was beyond the scope of the present project.

In summary, culturing ASCs in multilayer high-density sheets and stimulating them with inflammatory cytokines such as TNFα and IFNγ changes their expression of immunomodulatory genes and improves their ability to promote cell proliferation and angiogenesis as demonstrated by a reduction in *TNFA*, *IDO*, and *PTGS2* expression and augmentation of VEGFA secretion. Additionally, conditioned medium of these ASC-sheets enhanced fibroblast migration and endothelial cell proliferation in vitro, suggesting that using multilayer high density ASC-sheets cultured in the presence of TNFα and IFNγ are potentially the better choice for the treatment of injured or ischemic tissues.

## Electronic supplementary material

Supplementary fig. 1 Secretion of VEGFA, FGF2 and L-kynurenine from ASCs seeded in 4 different densities and cultured with or without 10 ng/ml TNFα/25 ng/ml IFNγ (low inflammatory) and 20 ng/ml TNFα +50 ng/ml IFNγ (high inflammatory) for 48 h. Each bar represents average concentration per ng DNA ± SD from 3 ASC donors in triplicate (*P < 0.05, **P < 0.01 when compared with control condition within same density, # *P* < 0.05, ## *P* < 0.01 when compared with control density (8000 cells/cm^2^) in same culture condition). DNA of ASCs was measured with Cyquant® cell proliferation assay. (GIF 2 kb)

High Resolution Image (TIFF 3192 kb)
