# Supplementary figures and images for: Effect of Cell Seeding Density and Inflammatory Cytokines on Adipose Tissue-Derived Stem Cells: an in Vitro Study
Source: Stem Cell Rev. 2017 Jan 24;13(2):267–77. doi: 10.1007/s12015-017-9719-3 (PMC5380713; doi:10.1007/s12015-017-9719-3)

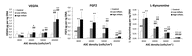

Supplement: Supplementary file 1 — Secretion of VEGFA, FGF2 and L-kynurenine from ASCs seeded in 4 different densities and cultured with or without 10 ng/ml TNFα/25 ng/ml IFNγ (low inflammatory) and 20 ng/ml TNFα +50 ng/ml IFNγ (high inflammatory) for 48 h. Each bar represents average concentration per ng DNA ± SD from 3 ASC donors in triplicate (*P < 0.05, **P < 0.01 when compared with control condition within same density, # P < 0.05, ## P < 0.01 when compared with control density (8000 cells/cm2) in same culture condition). DNA of ASCs was measured with Cyquant® cell proliferation assay. (GIF 2 kb) [file 12015_2017_9719_Fig7_ESM.gif]

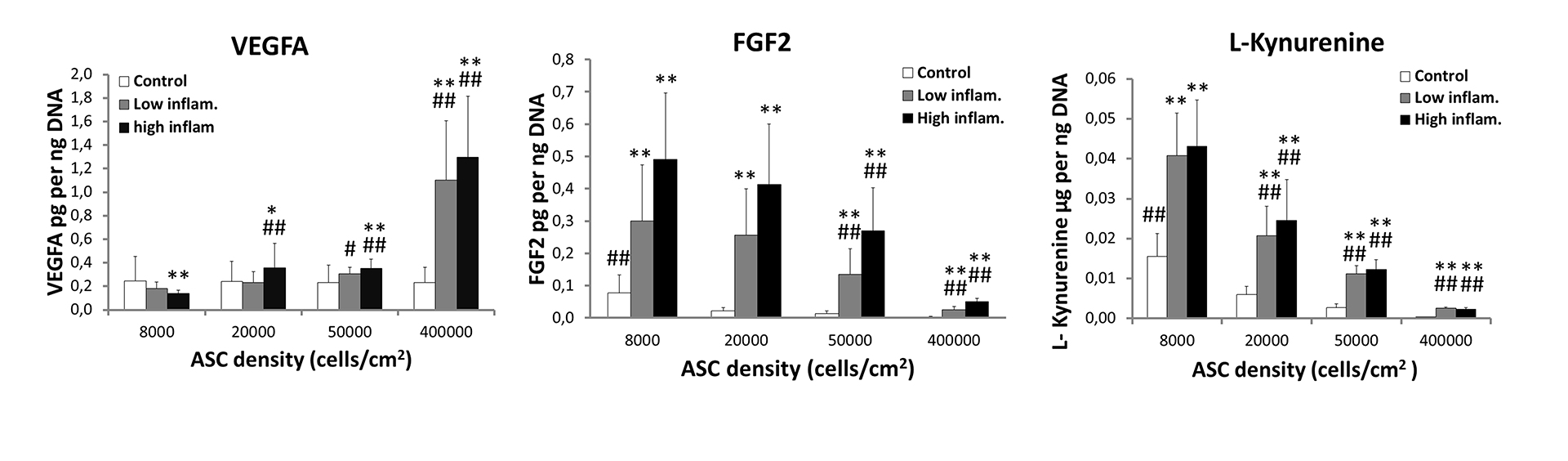

Supplement: Supplementary file 2 — High Resolution Image (TIFF 3192 kb) [file 12015_2017_9719_MOESM1_ESM.tif]
